# Investigation of 95 variants identified in a genome-wide study for association with mortality after acute coronary syndrome

**DOI:** 10.1186/1471-2350-12-127

**Published:** 2011-09-29

**Authors:** Thomas M Morgan, John A House, Sharon Cresci, Philip Jones, Hooman Allayee, Stanley L Hazen, Yesha Patel, Riyaz S Patel, Danny J Eapen, Salina P Waddy, Arshed A Quyyumi, Marcus E Kleber, Winfried März, Bernhard R Winkelmann, Bernhard O Boehm, Harlan M Krumholz, John A Spertus

**Affiliations:** 1 Department of Pediatrics, Vanderbilt University School of Medicine, Nashville, TN USA; 2 Saint Luke's Mid America Heart Institute and University of Missouri-Kansas City, Kansas City, MO USA; 3 Department of Internal Medicine, Cardiovascular Division, Washington University School of Medicine, St. Louis, MO USA; 4 Department of Preventive Medicine, USC Keck School of Medicine, Los Angeles, CA USA; 5 Center for Cardiovascular Diagnostics and Prevention, Department of Cell Biology, Lerner Research Institute, Cleveland Clinic, Cleveland, OH, USA; 6 Emory University School of Medicine, Atlanta, GA, USA; 7 Cardiff University, Cardiff, Wales, UK; 8 NINDS/NIH, Bethesda, MD, USA; 9 LURIC non profit LLC, Freiburg im Breisgau, Germany, and Mannheim Institute of Public Health, Medical Faculty Mannheim, University of Heidelberg, Germany; 10 synlab Services GmbH, Mannheim, and Mannheim Institute of Public Health, Medical Faculty Mannheim, University of Heidelberg, Germany, and Clinical Institute of Medical and Chemical Laboratory Diagnostics, Medical University of Graz, Austria; 11 Cardiology Group Sachenhausen, Frankfurt Sachsenhausen, Germany; 12 Division of Endocrinology, Diabetes and Metabolism, Graduate School of Molecular Diabetology and Endocrinology, Ulm University, Germany; 13Robert Wood Johnson Clinical Scholars Program and Department of Internal Medicine, Yale University School of Medicine, New Haven, CT USA

## Abstract

**Background:**

Genome-wide association studies (GWAS) have identified new candidate genes for the occurrence of acute coronary syndrome (ACS), but possible effects of such genes on survival following ACS have yet to be investigated.

**Methods:**

We examined 95 polymorphisms in 69 distinct gene regions identified in a GWAS for premature myocardial infarction for their association with post-ACS mortality among 811 whites recruited from university-affiliated hospitals in Kansas City, Missouri. We then sought replication of a positive genetic association in a large, racially diverse cohort of myocardial infarction patients (N = 2284) using Kaplan-Meier survival analyses and Cox regression to adjust for relevant covariates. Finally, we investigated the apparent association further in 6086 additional coronary artery disease patients.

**Results:**

After Cox adjustment for other ACS risk factors, of 95 SNPs tested in 811 whites only the association with the rs6922269 in *MTHFD1L *was statistically significant, with a 2.6-fold mortality hazard (*P *= 0.007). The recessive A/A genotype was of borderline significance in an age- and race-adjusted analysis of the entire combined cohort (N = 3095; *P *= 0.052), but this finding was not confirmed in independent cohorts (N = 6086).

**Conclusions:**

We found no support for the hypothesis that the GWAS-identified variants in this study substantially alter the probability of post-ACS survival. Large-scale, collaborative, genome-wide studies may be required in order to detect genetic variants that are robustly associated with survival in patients with coronary artery disease.

## Background

Genome-wide association studies (GWAS) have identified robust genetic associations in a variety of common diseases [1], including myocardial infarction (MI) [2-6]. The GWAS approach, with its emphasis on large sample sizes and inclusion of hundreds of thousands of genetic markers, has produced a degree of reproducibility that was generally lacking in earlier candidate gene studies of MI [7]. However, nine GWAS-identified genetic susceptibility markers, all meeting criteria for genome-wide statistical significance, collectively account for only 3% of the estimated heritability of early-onset myocardial infarction (MI), raising questions about the clinical utility of such markers for predicting MI [1].

In contrast to the identification of risk markers for incident disease, risk-stratifying patients with established disease, including those who have suffered an MI, is a cornerstone of modern cardiovascular care. Yet association studies of GWAS-generated candidate genes with prognosis following acute coronary syndromes (ACS), including unstable angina, non-ST elevation myocardial infarction (NSTEMI), and ST-elevation myocardial infarction (STEMI), require resource-intensive longitudinal designs and have rarely been performed. Although the post-ACS period is considered high risk, there is substantial heterogeneity in patient outcomes, and genetic markers could potentially be useful in defining risk, guiding treatment, and understanding the mechanisms of recurrent events and mortality. In contrast to genetic screening for incident MI, which requires screening large populations of patients without recognized disease, prognostically important genetic variations after an ACS could accelerate translation to clinical practice by focusing upon a narrower cohort of patients at high risk. Moreover, the identification of genetic pathways leading to a poor prognosis following ACS may identify new pathways of disease progression that could become novel targets for the chemoprevention of recurrent ACS.

GWAS-identified risk factors for incident MI pose an important opportunity to identify genetic markers of prognosis in an ACS population, given the clinical logic that a validated risk factor for MI occurrence may also lead to more rapid disease progression after an initial event. To test this possibility, we selected as a pool of candidate prognostic markers the 95 most statistically significant of approximately 2.5 million genetic variants tested in a GWAS of premature MI occurrence (Myocardial Infarction Genetics Consortium) [5]. We specifically tested the hypothesis that these risk markers for MI would be associated with all-cause mortality within 3 years following ACS.

## Methods

### Identification of Candidate Genes

We tested 95 SNPs in 63 individual genes, and an additional 6 distinct gene regions containing more than one genetic locus. The 95 candidate SNPs were ranked the most statistically significant (*P *< 1 × 10^-5^) of all ~2.5 million SNPs that were included on, or imputed from, the Affymetrix 6.0 microarray and brought forward into replication stage 3 of the Myocardial Infarction Genetics Consortium Study [5].

### Study Population and Genotyping

Our study design called for testing genetic markers for prognostic association with 3-year mortality in an ACS cohort, and attempting replication of significant associations in additional cohorts of MI and/or coronary artery disease (CAD) patients. The discovery cohort was comprised of 811 self-reported white patients of European ancestry with ACS who were identified from a consecutive series of patients presenting to two Kansas City, MO hospitals (Mid-America Heart Institute and Truman Medical Center), from March 2001 through June 2003. Standard definitions were used to diagnose ACS patients with either myocardial infarction or unstable angina [8,9]. Individuals were monitored for incident deaths from any cause, as determined by periodic queries of the Social Security Administration Death Master File [10]. Follow-up was planned for a minimum of 3-years.

The Translational Research Investigating Underlying disparities in acute Myocardial infarction Patients' Health status study (TRIUMPH) served as the replication cohort, as described in a recent publication [11]. This study recruited several sites that were enriched for African-American patients with MI and was specifically designed to address racial disparities in outcomes, including genetic variations between races. Patients were adults > 18 years of age with elevated cardiac biomarkers (troponin or creatine kinase-MB fraction) as well as other clinical evidence of MI (ECG ST-segment changes or prolonged ischemia signs/symptoms). Collected data included baseline chart abstractions for demographic and medical history, followed by study coordinators contacting patients for follow-up interviews at 1, 6, and 12 months after MI. Long-term mortality was assessed by periodic queries of the Social Security Administration Death Master File [10].

Additional validation cohorts for survival analysis involving one SNP, rs6922269 in the *MTHFD1L *gene, were provided by Cleveland Clinic GeneBank (coronary artery disease including acute myocardial infarction) [12,13], Emory Cardiology Biobank (cardiac catheterization patients) [14], and the Ludwigshafen Risk and Cardiovascular Health (LURIC) study (patients hospitalized for coronary angiography) [15]. Clinical details of the cohorts are described in Appendix A.

Genotyping of the 95 SNPs was performed at the Broad Institute at MIT using the iPLEX MassARRAY platform (Sequenom) on extracted leukocyte DNA (TRIUMPH) or whole genome amplified DNA (Mid-America Heart cohort) [16,17]. More extensive details of the cases, DNA extraction methods, and genotyping procedures have been recently described [5,7,18]. Flanking DNA sequence and other identifiers for each genetic variant are available upon request from the authors.

### Statistical Analysis

Genotype distributions were examined for significant deviations (*P *< 0.05) from Hardy-Weinberg equilibrium (HWE). Chi-square testing was used to screen for possible HWE violations, which were further investigated for statistically significant departure from HWE expectations by Monte Carlo testing involving 10, 000 random reshufflings of alleles [19].

Initially, Kaplan-Meier survival analysis was performed for each variant using SPSS 17.0 (Chicago, IL). The equality of survival curves was tested by the log-rank test pooled over all genotypic strata (one degree of freedom). If the log-rank *P *value was < 0.05, then pairwise log-rank tests were performed to explore which genotype or genotypes were most likely to confer mortality risk. Cox regression models were then used to adjust positive associations for age, sex, hypertension, ACS type, prior myocardial infarction, prior revascularization, congestive heart failure, diabetes, renal failure, marital status, educational level, menopause, and smoking and alcohol use prior to the ACS. We tested proportional-hazards assumption for each covariate using Shoenfeld residuals. We report raw P values and considered the conservative Bonferroni correction (0.05/95 = 0.0005) to represent the study-wide statistical significance threshold [20], but in addition to considering chance, we also considered canonical epidemiological principles of causation, including magnitude of effect, allelic dose-response, and adjustment for confounding.

Our sample had 93% power to detect an association, by the log rank test (*P *< 0.05), for a hazard ratio of 2.5 or higher, given a frequent genotype (0.5), and 80% power to detect a hazard ratio of 3.3 or higher, given an infrequent genotype (0.1) [21]. Given that genetic variants conferring more modest effects may not reach the conventional statistical significance level of *P *< 0.05, we sought to explore the possibility that null results might be related to lack of power (type II error), by examining the characteristics of the overall *P *value distribution by visualizing a Q-Q plot (quantile-quantile) using SPSS 17.0 (Chicago, IL).

## Results

The clinical characteristics of the 811 cases are described in Table 1. The population of ACS cases included 308 STEMI (38%), 284 NSTEMI (35%), and 219 unstable angina patients (27%). A family history of coronary artery disease or myocardial infarction among first-degree relatives was found in over half of cases (52%). In addition, cardiac risk factor profiles were typical of a population with ACS, with over one half of patients having hypercholesterolemia and hypertension, a third with history of smoking, and over one fifth with diagnosed diabetes. Previous revascularization had been performed in over a third of the cases.

**Table 1:** Characteristics of 811 White Subjects with Acute Coronary Syndrome at Baseline

Characteristic	Male ACS Cases (N = 550)	Female ACS Cases (N = 261)
Mean age in years (SD)	60.7 (12.5)	63.1 (13.2)

Mean body mass index (SD)	29.1 (5.5)	29.9 (6.9)

Family history of CAD/MI (%)	279 (50.7)	135 (51.7)

Prior myocardial infarction (%)	142 (25.8)	74 (28.4)

Prior revascularization (%)	205 (37.3)	83 (31.8)

Congestive heart failure (%)	23 (4.2)	18 (6.9)

Hypertension (%)	305 (55.5)	182 (69.7)

Diabetes Mellitus (%)	116 (21.1)	77 (29.5)

Hypercholesterolemia (%)	314 (57.1)	162 (62.1)

Postmenopausal (%)	--	189 (68.6)

College graduate (%)	166 (30.2)	40 (15.3)

Smoking < 30 days ago (%)	183 (33.3)	85 (32.6)

Alcohol frequency > 1/month (%)	221 (40.2)	38 (14.6)

Continuous variables are shown as mean (SD); categorical variables are number (%)

There were 90 deaths in the cohort, which was followed for a mean of 39.1 months, with maximum follow-up time of 60 months. Risk factor data were missing for 2 individuals. There were marked differences in overall cardiac risk factor profiles, as expected, with deceased patients having relatively advanced age, as well as more extensive cardiovascular co-morbidity, including congestive heart failure (Table 2).

**Table 2:** Clinical Characteristics of Surviving and Deceased Patients in the Follow-up Cohort

Characteristic	Survivors (N = 721)	Deceased (N = 90)
Mean age in years (SD)‡	60.5 (12.5)	69.5 (11.6)

Mean body mass index (SD)	29.4 (6.0)	28.8 (6.6)

Family history of CAD/MI (%)	374 (52.0)	40 (44.4)

Prior myocardial infarction(%)‡	176 (24.5)	40 (44.4)

Prior revascularization (%)‡	241 (33.5)	47 (52.2)

Congestive heart failure (%)‡	28 (3.9)	13 (14.4)

Hypertension (%)*	423 (58.8)	63 (70.0)

Diabetes Mellitus (%)‡	150 (20.9)	43 (47.8)

Hypercholesterolemia (%)	419 (58.3)	57 (63.3)

College graduate (%)	185 (25.9)	19 (21.6)

Smoking < 30 days ago (%)*	249 (34.7)	18 (20.0)

Alcohol frequency > 1/month (%)‡	348 (55.8)	19 (24.4)

Continuous variables are shown as mean (SD); categorical variables are number (%)

A total of 95 variants in 69 distinct genes or gene regions were genotyped. The average genotype call rate for these variants was 99.3%. Two assays failed (*FTO *rs9941349, *CETP *rs6499863). Eight variants violated HWE at the P < 0.05 level (additional file 1: Table S1).

**Figure 1:**
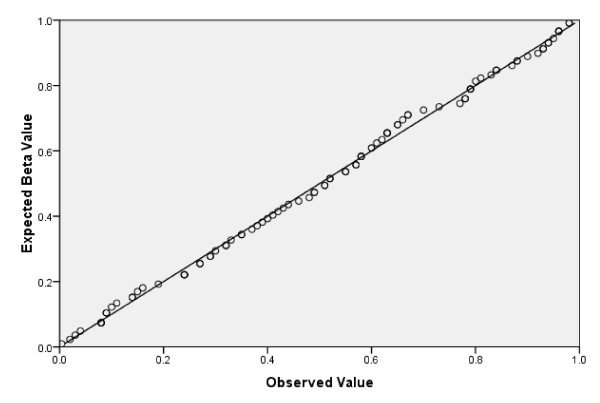
**Kaplan-Meier log-rank Q-Q Plot of for 95 Genetic Variants**.

The genotype distributions, numbers of deaths by genotypic category, and unadjusted *P *values for all 95 genetic variables are shown in additional file 1: Table S1, with the *P *value distributions summarized in the Q-Q plot shown in Figure 1. Overall, there were 4 positive associations (*P *< 0.05). Three of these associations did not retain statistical significance in a Cox proportional hazards adjusted model (rs6458545, *P *= 0.36; rs769449, *P *= 0.41; rs7754840, *P *= 0.13). However, the *MTHFD1L *association with mortality hazard remained statistically significant following adjustment for traditional cardiac risk factors. Of these covariates, age (HR 1.04/year; *P *< 0.001), diabetes (HR 3.2; *P *< 0.001), prior myocardial infarction (HR 1.7; *P *= 0.04), congestive heart failure (2.0; *P *= 0.04), and unmarried status (HR 1.3; *P *= 0.003) had significant associations with mortality when modeled independent of genotype. The A/A genotype (versus G/G reference) of the mitochondrial-expressed *MTHFD1L *(methylenetetrahydrofolate dehydrogenase (NADP+ dependent) 1-like) gene variant rs6922269 was associated with hazard ratio of 2.6 (*P *= 0.007). The pairwise Kaplan-Meier analysis showed significant differences for the comparison of A/A vs G/G (*P *= 0.01), and A/G vs G/G (*P *= 0.02), but not A/A vs A/G (*P *= 0.35), implicating the A allele as a risk factor. Of the 12 A/A individuals who died (50% female), frequent co-morbidities were hypertension (N = 9), current or former smoking (N = 9), no current alcohol consumption (N = 9), hyperlipidemia (N = 8), family history of coronary artery disease (N = 8), and type 2 diabetes (N = 7). However, the frequencies of such co-morbidities were not significantly different from the 78 other deceased patients with any other *MTHFD1L *rs6922269 genotype.

**Figure 2:**
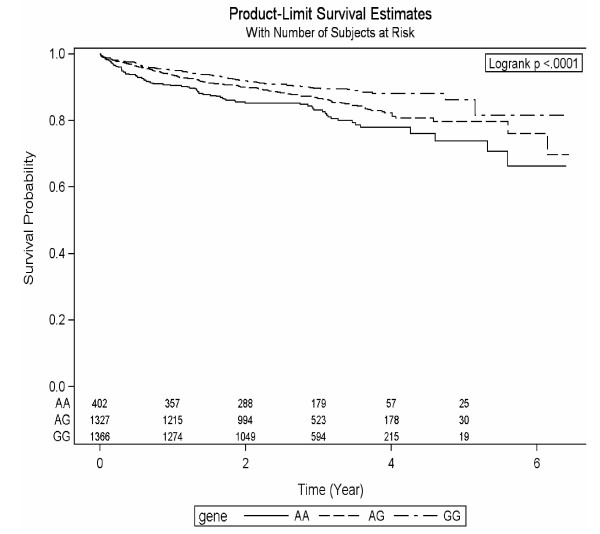
**Kaplan-Meier Survival Curve for *MTHFD1L *rs6922269 (N = 3095)**.

In order to limit multiple comparisons, only the *MTHFD1L *rs6922269 variant was brought forward for additional genotyping in the TRIUMPH cohort. In order to maximize power, the entire group of patients was subjected to combined Kaplan-Meier and Cox regression analysis. Characteristics, by genotype, of the entire study group appear in Table 3. As expected, mean follow-up time did not significantly differ by genotype. The A/A genotype of rs6922269 was strongly associated with all-cause mortality in the entire group (Figure 2; *P *< 0.0001). However, after adjustment for age, sex, and race, the hazard ratio for mortality in association with the A/A genotype was 1.35, with a borderline statistically significant P value of 0.052. Although sample size was too limited to document any formal trend towards increased mortality risk by number of A alleles, a suggestive decrement in risk was observed in Cox-adjusted hazard ratios, from 1.35 (A/A), to 1.21 (A/G) to 1.0 (G/G reference), as shown in Table 4. In the TRIUMPH replication cohort alone (N = 2284), a striking racial dichotomy was noted, with the lowest survival rate (81%) observed in African-American A/A homozygotes (additional file 1, Table S2), compared with 90% or greater survival in all other genotype categories for Whites and African-Americans. The difference in survival by genotype was statistically significant in African-Americans (*P *= 0.015) but not in Whites (*P *= 0.284).

**Table 3:** Characteristics of patients by *MTHFD1L *genotype (N = 3095).

*Outcome*		GG (n = 1366)	AG (n = 1327)	AA (n = 402)	*P *Value
Patient Died	395 (12.8%)	137 (10.0%)	182 (13.7%)	76 (18.9%)	< 0.001

Time (Year)					0.120
Mean ± SD	2.8 ± 1.2	2.8 ± 1.1	2.7 ± 1.2	2.8 ± 1.4	
Median (IQR*)	2.7 (2.0, 3.6)	2.8 (2.1, 3.7)	2.6 (2.0, 3.6)	2.8 (1.8, 3.6)	

*Demographics and History*					

Age	59.3 ± 12.5	59.9 ± 12.2	59.1 ± 12.8	57.9 ± 12.4	0.010

Sex					0.003
Male	2095 (67.7%)	944 (69.1%)	909 (68.5%)	242 (60.2%)	
Female	1000 (32.3%)	422 (30.9%)	418 (31.5%)	160 (39.8%)	

Race Category					< 0.001
White/Caucasian	2315 (74.9%)	1189 (87.3%)	943 (71.1%)	183 (45.6%)	
Black/African American	607 (19.7%)	109 (8.0%)	303 (22.9%)	195 (48.6%)	
Other	167 (5.4%)	64 (4.7%)	80 (6.0%)	23 (5.7%)	
Unknown	6	4	1	1	

*IQR: interquartile range

**Table 4:** Cox proportional hazards analysis involving all patients (N = 3095).

Variable	Hazard ratio	95% confidence interval	Chi-Square *P *
Age	1.05	(1.04-1.05)	< .0001

Male sex	1.05	(0.85-1.29)	0.66

Black race	2.56	(2.05-3.20)	< .0001

*MTHFD1L *A/A*	1.35	(1.00-1.82)	0.05

*MTHFD1L *A/G*	1.21	(0.97-1.52)	0.09

*Reference genotype G/G

Given the equivocal, but nominally statistically significant, results in our primary replication cohort, we collaborated with independent investigators to determine the extent to which our findings might be replicable in other cohorts of patients with CAD. Raw survival data are shown in Table 5. The overall proportion of deaths in all patients combined, by genotype, was essentially the same across all studies. Table 3 (additional file 1) presents the A allele frequency of the rs6922269 SNP of *MTHFD1L*, showing high similarity of race-specific allele frequencies across sites. Each site performed a standardized Cox proportional hazards analysis, stratified by race (if applicable) and adjusted for age and sex. The A/A genotype was not associated with mortality in any of the 3 independent cohorts. In the Emory cohort, among African-Americans with CAD, the A/A genotype hazard ratio (compared to G/G reference) was 1.126 (95% CI, 0.458 - 2.770; p = 0.796). Among whites, the HR was 0.684 (95% CI, 0.297 - 1.578; p = 0.684). Similar results for whites were obtained in the Cleveland Clinic cohort (A/A = 0.834 (95% CI, 0.402 - 1.728; p = 0.624) as well as the LURIC cohort (A/A = 0.846 (95% CI, 0.657 - 1.090; p = 0.196), which had the longest follow-up of any study, with a median follow-up of 10.1 years. The corresponding median follow-up time in the Emory cohort was 2.4 years, and for Cleveland Clinc, it was 3 years.

**Table 5:** Mortality, by MTHFD1L rs6922269 genotype, in all CAD cohorts (N = 9181).

cohort	race	G/G deaths	N	%	A/G deaths	N	%	A/A deaths	N	%
MAHI*	White	57	792	7.2	43	640	6.7	4	118	3.4

	AA†	11	127	8.8	29	307	9.4	29	166	17.5

Emory	White	68	927	7.3	49	654	7.5	6	110	5.5

	AA	8	106	7.5	17	221	7.7	12	132	9.1

Cleveland	White	76	1281	5.9	69	918	7.5	8	145	5.5

LURIC	White	489	1592	30.7	362	1227	29.5	72	219	32.9

Total		709	4825	14.7	569	3967	14.3	131	890	14.7

*Mid-America Heart Institute; †African-American

## Discussion

We initially found a nominally statistically significant association between the A/A genotype of *MTHFD1L *and mortality in an initial cohort of 811 white patients with ACS. However, in the primary replication cohort, as well as three additional replication cohorts of patients with MI and/or CAD, this association was not confirmed, with a total of over 9, 000 patients studied.

Our experience in this study highlights that robust replication in multiple independent studies is a critical criterion for judging the validity of genetic associations. However, assembling the clinical data required to perform such replication studies is challenging, given that it may require not only the ascertainment of many thousands of patients, but also oversampling for minorities (by race and/or sex), and then tracking outcomes for many years. Our report of survival data for multiple high-priority SNPs advances the relatively neglected field of post-ACS genetic prognosis.

Although chance is the likely explanation for the apparently statistically significant association that we initially observed between mortality and rs6922269, other epidemiological factors should be considered in judging the cause-effect relationship between a putative risk factor and an outcome. In particular, prior evidence that the A allele is a risk factor for myocardial infarction is equivocal. Two major independent GWAS have reported the A allele of *MTHFD1L *to be associated with early-onset myocardial infarction [5,22]. First, the Wellcome Trust Case Control Consortium (WTCCC) reported a per-allele risk of 1.23 [1.15-1.33] for the A allele of rs6922269 and coronary artery disease, with a genome-wide statistical significance of 2.90 × 10^-8 ^[22]. Subsequently, the transatlantic Coronary ARtery DIsease Genome wide Replication and Meta-analysis (CARDIoGRAM) consortium performed a meta-analysis of 14 GWAS studies (22, 233 cases, 64, 763 controls), and *MTHFD1L *A risk allele not among the statistically significant genome-wide associations (*P *= 7.38 × 10-5; A frequency 0.28) [23]. The A allele was not associated with the presence of significant coronary arterial stenosis in a series of consecutive patients referred for coronary angiography due to known or suspected stable CAD [24].

Clearly, even findings that meet accepted genome-wide criteria for statistical significance in individual studies should be replicated widely before genetic associations are accepted as valid and robust. In addition, in light of our study, it appears that realistic sample size calculations for prognostic studies may need to posit effect sizes at least as small as those that have emerged from GWAS studies of the occurrence of CAD, which are typically substantially less than 1.4. The challenge of mustering sufficient power is further amplified in prognosis studies by the fact that only a small proportion of patients will experience mortality within several years of follow-up. Therefore, sample size requirements for prognostic studies are expected to be at least as large as those for case-control studies. While it is beyond the scope of the present study to prescribe detailed sample size recommendations for future studies of prognosis in patients with common cardiovascular diseases, we would note that the CARDIoGRAM consortium included 22, 233 patients with CAD, with an approximate 3:1 control-to-case ratio. In addition to requiring large sample sizes, considerations for oversampling clinically relevant subgroups, outcome adjudication, and genotyping scope and methods are all emerging challenges to field of cardiovascular genetics as it seeks to leverage genetic risk factors to better risk-stratify outcomes in AMI suvivors. Additional limitations to this study include the heterogeneity of the present study, with respect to rs6922269 and our inability to discern whether or not there could be an effect in particular subgroups of patients, given the overall absence of a significant association.

## Conclusions

We found no convincing support for the hypothesis that SNPs identified from GWAS studies of cardiac risk are associated with all-cause mortality following ACS, suggesting that independent GWAS studies of cohorts of many thousands of ACS patients may be required in order to identify prognostic factors in biological pathways promoting post-ACS mortality.

## Competing interests

The authors declare that they have no competing interests.

## Authors' contributions

TMM, HK, and JAS conceived of the study, and participated in its design and coordination and drafted the manuscript. TMM and SC performed genotyping for the primary study. JH and PJ participated in the design of the study and performed the statistical analysis. HA, SLH, YP, RSP, DJE, SPW, AAQ, MK, WM, BRW, and BOB executed all aspects of the replication studies and contributed data, statistical analysis, and critical input into the drafting of the manuscript. All authors read and approved the final manuscript.

## Pre-publication history

The pre-publication history for this paper can be accessed here:

http://www.biomedcentral.com/1471-2350/12/127/prepub

## Supplementary Material

Additional file 1 **Tables S1-S3**. Description of additional study populations. Genotyping methods in additional study populations. Supplemental results in additional study populations. Supplemental Table 1. Supplemental Table 2. Supplemental Table 3. Click here for file
