# Supplementary material for: Investigation of 95 variants identified in a genome-wide study for association with mortality after acute coronary syndrome
Source: BMC Med Genet. 2011 Sep 29;12:127. doi: 10.1186/1471-2350-12-127 (PMC3190329; doi:10.1186/1471-2350-12-127)
Supplement: Additional file 1 — Tables S1-S3. Description of additional study populations. Genotyping methods in additional study populations. Supplemental results in additional study populations. Supplemental Table 1. Supplemental Table 2. Supplemental Table 3. [file 1471-2350-12-127-S1.RTF]

Supplemental methods and results with description of validation cohorts for survival analysis involving one SNP, rs6922269 in the MTHFD1L gene, provided by Cleveland Clinic GeneBank (coronary artery disease including acute myocardial infarction), Emory Cardiology Biobank (cardiac catheterization patients), and the Ludwigshafen Risk and Cardiovascular Health (LURIC) study (patients hospitalized for coronary angiography).

Study populations
Cleveland Clinic: GeneBank is a single-site repository generated from patients undergoing elective diagnostic coronary angiography or elective cardiac computed tomographic angiography with extensive clinical and laboratory characterization and longitudinal observation.  Ethnicity was self-reported and information regarding demographics, medical history, and medication use was obtained by patient interviews and confirmed by chart reviews.  All clinical outcome data were verified by source documentation.  CAD was defined as adjudicated diagnoses of stable or unstable angina, MI (adjudicated definition based on defined electrocardiographic changes or elevated cardiac enzymes), angiographic evidence of ≥ 50% stenosis of one or more major epicardial vessel, and/or a history of known CAD (documented MI, CAD, or history of revascularization).  Prospective cardiovascular risk was assessed by the incidence of major adverse cardiac events (MACE) during three years of follow-up from the time of enrollment, which included nonfatal MI, nonfatal stroke, and all-cause mortality.  Adjudicated outcomes ascertained over the ensuing 3 years for all subjects following enrollment were confirmed using source documentation.  All patients provided written informed consent prior to being enrolled in GeneBank and the study was approved by the Institutional Review Board of the Cleveland Clinic.  

Emory:  In 2150 Caucasian and African American participants, recruited from the Emory Cardiology Biobank that enrolled consecutive patients undergoing cardiac catheterization between 2003 and 2010, we documented demographic characteristics, medical histories, and behavioral factors.  Specific details regarding disease and risk factor definitions and other phenotyping have been described previously.(Patel, Su et al.)  Patients with heart transplantation and missing or incomplete phenotype or genotype data were excluded. All subjects were prospectively followed by telephone interview, chart abstraction and through state vital records data to document major events and all cause death.  Follow-up was performed at time periods ranging from 1-5 years. The study was approved by the Institutional Review Board and all subjects provided written informed consent. 

LURIC:  The Ludwigshafen Risk and Cardiovascular Health (LURIC) study includes consecutive white patients hospitalized for coronary angiography between June 1997 and May 2001. A detailed description of LURIC has been published (1). The study was approved by the ethics review committee at the “Landesärztekammer Rheinland-Pfalz” (Mainz, Germany). Written informed consent was obtained from each of the participants. Clinical indications for angiography were chest pain or non-invasive tests consistent with myocardial ischemia. To limit clinical heterogeneity, individuals suffering from acute illness other than acute coronary syndromes, chronic non-cardiac diseases and a history of malignancy within the five past years were excluded.
	

Genotyping
Cleveland Clinic:  As part of ongoing studies in GeneBank, 3031 subjects were genotyped on the Affymetrix Genome-Wide Human SNP Array V6.0.  Complete genotype data for rs6922269 and longitudinal mortality outcomes were available in 2345 subjects with documented CAD.  Samples analyzed by the 6.0 arrays yielded an average call rate by the Birdseed algorithm of 99.3%.

Emory:  Genotyping was performed with the SNPstream (Beckman Coulter) platform at Emory University, Atlanta, GA. (Bell, Chaturvedi et al. 2002; Kutyavin, Milesi et al. 2006) The genotyping rate for the rs6922269 SNP was 98%.  The GenomeLab SNPstream Genotyping System Software Suite v2.3 (Beckman Coulter, Inc., Fullerton, CA) was used for array imaging and genotype calling. To ensure genotyping accuracy and reproducibility two internal quality control samples were included and each run in triplicate, on each of the 384-well arrays.

LURIC:  The rs6922269 polymorphism was genotyped in LURIC by using microarray chips (Genome-wide Human SNP Array 6.0 (Affymetrix) and 200k Cardio-Metabochip (Illumina)). 


Results

Cleveland Clinic GeneBank Summary for rs6922269 survival analysis
Median follow up for this genotyped subset (all whites) is 1095 days;
All Whites mean age 61.54 (11.06); 74.29% male
Total n= 2345
Total deaths (all-cause) = 153
GG - 1281 (76)
AG - 919 (69)
AA - 145 (8)
Kaplan Meier Log rank p-value = 0.158
Cox Survival HR adjusted for age and gender
Genotypic HR
GG = 1 (ref)
AG = 1.276 (95% CI, 0.921 – 1.768; p=0.143)
AA = 0.834 (95% CI, 0.402 – 1.728; p=0.624)
Allelic HR (1.082 (0.842 – 1.390); p=0.539)

Emory Cardiology Biobank Summary for rs6922269 survival analysis
Median follow up for this genotyped subset (white and AA) is 891 days; 65.8% male; mean age 61.5 (10.28); 21.3% AA
Whites (median f/u 901 days; mean age 62.46(9.87); male 69.2%) 
Total n= 1691
Total deaths (all-cause) = 123
GG - 927 (68)
AG - 654 (49)
AA - 110 (6)
Kaplan Meier Log rank p-value = 0.754
Cox Survival HR adjusted for age and gender
Genotypic HR
GG = 1 (ref)
AG = 0.978 (95% CI, 0.677 – 1.413; p=0.978)
AA = 0.684 (95% CI, 0.297 – 1.578; p=0.684)
Allelic HR (0.906 (0.677 – 1.213); p=0.508)

African Americans (median f/u 823 days; mean age 58.31(10.99); male 53.3%)
Total n= 459
Total deaths (all-cause) = 37
GG - 106 (8)
AG - 221 (17)
AA - 132 (12)
Kaplan Meier Log rank p-value = 0.873
Cox Survival HR adjusted for age and gender
Genotypic
GG = 1 (ref)
AG = 0.907 (95% CI, 0.390 - 2.110; p=0.821)
AA = 1.126 (95% CI, 0.458 - 2.770; p=0.796)
Allelic HR (1.078 (0.679 – 1.712); p=0.751)

LURIC: Summary for rs6922269 survival analysis
Whites (median f/u 10.1 years; mean age 62.67 (10.68); male 69.5%) 
Total n= 3038
Total deaths (all-cause) = 923
GG - 1592 (52.4%)
AG - 1227 (40.4%)
AA - 219 (7.2%)
Kaplan Meier Log rank p-value = 0.91
Cox Survival HR adjusted for age and gender
Genotypic HR
GG = 1 (ref)
AG = 0.921 (95% CI, 0.719 – 1.180; p=0.516)
AA = 0.846 (95% CI, 0.657 – 1.090; p=0.196)
Allelic HR (0.983 (0.886 – 1.091); p=0.746)

Supplemental Tables


Supplemental Table 1. Kaplan-Meier Analysis of Mortality post-ACS for 95 genetic variants.
Gene/SNP	Genotype	Number	Deaths (%)	P	Gene/SNP	Genotype	Number	Deaths (%)	P	
ANGPTL4	AA	1	0 (0.0)	.95	MC4R	CC	37	3   (8.1)	.46	
ANGPTL_E40K	AG	24	3 (12.5)		rs17782313	CT	284	30 (10.6)		
	GG	782	86 (11.0)			TT	483	57 (11.8)		
APOE	GG	81	10 (12.3)	.81	ITPR1
BHLHB2	AA	239	22   (9.2)	.92	
rs10402271	GT	348	35 (10.1)		rs1867000	AG	399	53 (13.3)		
	TT	373	43 (11.5)			GG	167	15   (9.0)		
CILP2	CC	607	67 (11.0)	.66	KIF6	CC	108	14 (13.0)	.83	
rs10415849	CT	183	18 (9.8)		rs20455	CT	377	39 (10.3)		
	TT	10	1 (10.0)			TT	322	37 (11.5)		
KLF14	AA	189	21 (11.1)	.49	gene desert	AA	77	10 (13.0)	.77	
rs10954284	AT	405	40 (9.9)		rs2943634	AC	353	39 (11.0)		
	TT	207	27 (13.0)			CC	374	41 (11.0)		
PCSK9	CC	23	(0.0)	.62	LPL	CC	648	74 (11.4)	.60	
rs11206510	CT	212	26 (12.3)		rs328	CG	146	15 (10.3)		
	TT	572	63 (11.0)			GG	12	  1  (8.3)		
FTO*	CC	273	29 (10.6)	.58	LDLR	AA	39	5 (12.8)	.27	
rs1121980	CT	365	45 (12.3)		rs3786722	AC	289	37 (12.8)		
	TT	165	14 (8.5)			CC	477	48 (10.1)		
XKR6	CC	154	22 (14.3)	.37	ABCA1	AA	21	  1 (4.8)	.79	
rs11774572	CT	392	39 (9.9)		rs3890182	AG	180	22 (12.2)		
	TT	259	28 (10.8)			GG	604	67 (11.1)		
TIMD4	CC	97	10 (9.7)	.94	DSC3	AA	346	33 (9.5)	.09	
rs1501908	CG	367	42 (11.4)		rs4398167	AT	363	42 (11.6)		
	GG	332	36 (10.8)			TT	95	15 (15.8)		
HNF4A	CC	782	85 (10.9)	.79	IGF2BP2	GG	379	42 (11.1)	.24	
rs16988929	CT	15	2 (13.3)		rs4402960	GT	332	43 (13)		
	TT	1	(0.0)			TT	85	3 (3.5)		
CILP2	GG	675	76 (11.3)	.44	ZNF366	CC	29	2 (6.9)	.88	
rs16996148	GT	125	12 (9.6)		rs4703910	CT	250	30 (12.0)		
	TT	4	0 (0.0)			TT	525	58 (11.0)		
TBL2	CC	625	74 (11.8)	.14	APOB	CC	538	59 (11.0)	.57	
rs17145738	CT	169	15 (8.9)		rs562338	CT	238	25 (10.5)		
	TT	11	0 (0.0)			TT	27	5 (18.5)		
SMAD3	CC	68	5 (7.4)	.63	PHACTR1	CC	109	10 (9.2)	.03	
rs17228212	CT	352	42 (11.9)		rs6458545	CG	365	31 (8.5)		
	TT	386	42 (10.9)			GG	319	46 (14.4)		
CXCL12	CC	604	67 (11.1)	.96	SORT1	AA	484	53 (11.0)	.67	
rs1746048	CT	179	19 (10.6)		rs646776	AG	287	31 (10.8)		
	TT	16	2 (12.5)			GG	33	5 (15.2)		
LIPC	CC	503	58 (11.5)	.60	APOB	GG	468	47 (10.0)	.79	
rs1800588	CT	260	26 (10.0)		rs6544366	GT	290	41 (14.1)		
	TT	43	5 (11.6)			TT	50	2 (4.0)		
CETP	CC	599	65 (10.9)	.96	HNF1A*	AA	117	10 (8.5)	.42	
rs289742	CG	181	20 (11.0)		rs7310409	AG	261	30 (11.5)		
	GG	22	2 (9.1)			GG	414	48 (11.6)		
SLC22A21	CC	617	69 (11.2)	.78	HAAO	AA	458	58 (12.7)	.08	
rs3127575	CT	166	18 (10.8)		rs7583085	AG	306	30 (9.8)		
	TT	22	2 (9.1)			GG	41	2 (4.9)		
CETP*	GG	370	37 (10.0)	.87	GCKR	AA	120	14 (11.7)	.19	
rs3764261	GT	327	42 (0.3)		rs780094	AG	366	47 (12.8)		
	TT	100	7 (7.0)			GG	321	29 (9.0)		
SLC22A21	AA	23	3 (13.0)	.84	TCF7L2	CC	402	47 (11.7)	.32	
rs3798156	AG	188	19 (10.1)		rs7903146	CT	331	39 (11.8)		
	GG	596	67 (11.2)			TT	71	4 (5.6)		
HMGCR	CC	316	37 (11.7)	.35	C11orf66
SYT7	CC	7	1 (14.3)	.24	
rs3846663	CT	362	35 (9.7)		rs7947046	CT	133	10 (7.5)		
	TT	116	11 (9.5)			TT	668	79 (11.8)		
MIA3	CC	613	70 (11.4)	.40	FOXP1	GG	183	19 (10.4)	.93	
rs4240934	CT	180	   19 (10.6)		rs9878602	GT	381	45 (11.8)		
	TT	12	   0 (0.0)			TT	237	26 (11.0)		
Chr 5*	AA	23	   1 (4.3)	.80	FTO*	AA	148	11 (7.4)	.49	
rs4242231	AG	182	  24 (13.2)		rs9939609	AT	360	47 (13.1)		
	GG	601	64 (10.6)			TT	296	32 (10.8)		
WDR12 ALS2CR8	AA	603	70 (11.6)	.38	CETP	AA	122	16 (13.1)	.61	
rs4675310	AG	187	18 (9.6)		rs9989419	AG	376	40 (10.6)		
	GG	15	  1 (6.7)			GG	300	33 (11.0)		
EDNRA	AA	9	  1 (11.1)	.48	WFS1	AA	130	16 (12.3)	.93	
rs4835412	AG	179	17 (9.5)		rs10010131	AG	371	40 (10.8)		
	GG	617	71 (11.5)			GG	302	34 (11.3)		
GALNT2	AA	313	33 (10.5)	.39	CHRNA5*	CC	363	35 (9.6)	.35	
rs4846914	AG	366	37 (10.1)		rs1051730	CT	338	43 (12.7)		
	GG	128	19 (14.8)			TT	105	12 (11.4)		
LIPG	CC	563	66 (11.7)	.16	NOTCH2	GG	638	78 (12.2)	.08	
rs4939883	CT	214	20 (9.3)		rs10923931	GT	155	11 (7.1)		
	TT	26	1 (3.8)			TT	13	1 (7.7)		
CDKN2B	AA	179	23 (12.8)	.30	PCSK9	GG	780	90 (11.5)	.09	
rs4977574	AG	388	42 (10.8)		rs11591147	GT	25	0 (0.0)		
	GG	238	23 (9.7)			TT	0	0 (0.0)		
CXCL12	AA	604	68 (11.3)	.94	SLC30A8	CC	402	45 (11.2)	.96	
rs501120	AG	179	19 (10.6)		rs13266634	CT	322	35 (10.9)		
	GG	15	2 (13.3)			TT	82	10 (12.2)		
PRNPIP	CC	456	57 (12.5)	.10	CHRNA5	AA	105	12 (11.4)	.33	
rs6429535	CT	291	28 (9.6)		rs16969968	AG	333	42 (12.6)		
	TT	58	4 (6.9)			GG	368	35 (9.5)		
LDLR	GG	646	75 (11.6)	.52	TRIB1	AA	249	25 (10.0)	.11	
rs6511720	GT	152	12 (7.9)		rs17321515	AG	386	38 (9.8)		
	TT	9	2 (22.2)			GG	167	26 (15.6)		
APOA5	AA	694	76 (11)	.98	FADS	CC	82	11 (13.4)	.41	
rs6589566	AG	107	13 (12.1)		rs174547	CT	363	32 (8.8)		
	GG	5	0 (0)			TT	354	46 (13.0)		
APOB	CC	205	21 (10.2)	.63	MIA3	AA	72	5 (6.9)	.24	
rs693	CT	392	43 (11)		rs17465637	AC	328	36 (11.0)		
	TT	204	24 (11.8)			CC	406	49 (12.1)		
TCF1	AA	285	33 (11.6)	.58	LCAT	AA	7	0 (0.0)	.52	
rs7953249	AG	377	38 (10.1)		rs2271293	AG	175	19 (10.9)		
	GG	140	14 (10)			GG	604	71 (11.8)		
IL6R	AA	280	29 (10.4)	.55	MVK	CC	186	23 (12.4)	.57	
rs8192284	AC	381	40 (10.5)		rs2338104	CG	390	34 (8.7)		
	CC	124	16 (12.9)			GG	225	31 (13.8)		
KCNE2	AA	575	66 (11.5)	.51	CRP	CC	67	7 (10.4)	.29	
rs973754	AG	210	21 (10)		rs2808630	CT	324	42 (13)		
	GG	20	2 (10)			TT	410	39 (9.5)		
KCNE2	CC	585	64 (10.9)	.73	TCF2	AA	210	25 (11.9)	.65	
rs9982601	CT	200	23 (11.5)		rs4430796	AG	401	39 (9.7)		
	TT	19	1 (5.3)			GG	189	25 (13.2)		
KDR	CC	769	86 (11.2)	.90	DSC3	AA	347	34 (9.8)	.14	
rs10014689	CT	38	4 (10.5)		rs4553696	AT	360	40 (11.1)		
	TT	1	0 (0)			TT	96	15 (15.6)		
FUT8	AA	736	83 (11.3)	.67	ADAMTS9	CC	464	54 (11.6)	.84	
rs10483778	AC	71	7 (9.9)		rs4607103	CT	291	28 (9.6)		
	CC	0	0 (0)			TT	47	8 (17.0)		
FUT8	AA	729	19 (10.6)	.79	CETP	CC	475	56 (11.8)	.65	
rs10483782	AG	66	0 (0)		rs4783962	CT	287	27 (9.4)		
	GG	5	1 (4.3)			TT	44	6 (13.6)		
ANGPTL3	AA	334	24 (13.2)	.24	
WDR12
ALS2CR8	CC	16	1 (6.3)	.55	
rs10889353	AC	376	64 (10.6)		rs6725887	CT	183	19 (10.4)		
	CC	97	70 (11.6)			TT	604	69 (11.4)		
LDLR	GG	477	18 (9.6)	.29	MTHFD1L	AA	72	12 (16.7)	.004	
rs1122608	GT	287	1 (6.7)		rs6922269	AG	314	42 (13.4)		
	TT	38	1 (11.1)			GG	416	34 (8.2)		
PHACTR1	CC	356	17 (9.5)	.08	MSRA	CC	437	53 (12.1)	.43	
rs12526453	CG	359	71 (11.5)		rs6995374	CG	324	24 (7.4)		
	GG	88 	33 (10.5)			GG	44	13 (29.5)		
CAMK1D/
CDC23	AA	547	37 (10.1)	.32	THADA	CC	9	0 (0.0)	.96	
rs12779790	AG	231	19 (14.8)		rs7578597	CT	161	20 (12.4)		
	GG	26	66 (11.7)			TT	637	70 (11)		
C11orf66
SYT7	CC	756	20 (9.3)	.88	APOE*	AA	23	0 (0.0)	.02	
rs12802349	CT	50	1 (3.8)		rs769449	AG	170	13 (7.6)		
	TT	0	23 (12.8)			GG	611	76 (12.4)		
CDKN2B	CC	235	42 (10.8)	.27	CDKAL1	CC	84	5 (6.0)	.04	
rs1333049	CG	388	23 (9.7)		rs7754840	CG	366	37 (10.1)		
	GG	183	68 (11.3)			GG	340	46 (13.5)		
LPA	AA	73	19 (10.6)	.98	JAZF1	AA	225	27 (12.0)	.58	
rs1367211	AG	330	2 (13.3)		rs864745	AG	395	43 (10.9)		
	GG	403	57 (12.5)			GG	182	19 (10.4)		
FTO*	CC	156	28 (9.6)	.70	KIAA1217	CC	485	59 (12.2)	.51	
rs1421085	CT	363	4 (6.9)		rs867040	CG	272	23 (8.5)		
	TT	288	75 (11.6)			GG	49	7 (14.3)		
LPL	GG	649	12 (7.9)	.78						
rs17482753	GT	147	2 (22.2)							
	TT	12	76 (11)							
*HWE deviation (P < 0.05); 1SLC22A2, SLC22A3, LPAL2, and LPA are near this SNP


Supplemental Table 2. Kaplan-Meier Analysis of Mortality post-ACS in TRIUMPH (N = 2284)
Gene/SNP	MTHFD1L Genotype	Number	Deaths (Survival%)	P	
All Patients	AA	284	33 (88)	0.056	
	AG	947	72 (92)		
	GG	919	68 (92)		
White Patients	AA	118	4 (96)	0.284	
	AG	640	43 (93)		
	GG	792	57 (92)		
African-American Patients	AA	166	29 (81)	0.015	
	AG	307	29 (90) 		
	GG	127	11 (91)		


Supplemental Table 3. MTHFD1L A frequencies
cohort	White	African-American	
MAHI	0.28	0.53	
Emory	0.26	0.53	
Cleveland	0.26	                    n/a	
LURIC	                0.27	                   n/a	
